# Tissue Engineered Bands of Büngner for Accelerated Motor and Sensory Axonal Outgrowth

**DOI:** 10.3389/fbioe.2020.580654

**Published:** 2020-11-20

**Authors:** Kate V. Panzer, Justin C. Burrell, Kaila V. T. Helm, Erin M. Purvis, Qunzhou Zhang, Anh D. Le, John C. O’Donnell, D. Kacy Cullen

**Affiliations:** ^1^Center for Brain Injury and Repair, Department of Neurosurgery, Perelman School of Medicine, University of Pennsylvania, Philadelphia, PA, United States; ^2^Center for Neurotrauma, Neurodegeneration and Restoration, Corporal Michael J. Crescenz Veterans Affairs Medical Center, Philadelphia, PA, United States; ^3^Department of Bioengineering, School of Engineering and Applied Science, University of Pennsylvania, Philadelphia, PA, United States; ^4^Department of Neuroscience, Perelman School of Medicine, University of Pennsylvania, Philadelphia, PA, United States; ^5^Department of Oral and Maxillofacial Surgery, School of Dental Medicine, University of Pennsylvania, Philadelphia, PA, United States; ^6^Department of Oral and Maxillofacial Surgery, Penn Medicine Hospital of University of Pennsylvania, Philadelphia, PA, United States

**Keywords:** tissue engineering, peripheral nervous system, Schwann cells, axon guidance, stem cells

## Abstract

Following peripheral nerve injury comprising a segmental defect, the extent of axon regeneration decreases precipitously with increasing gap length. Schwann cells play a key role in driving axon re-growth by forming aligned tubular guidance structures called bands of Büngner, which readily occurs in distal nerve segments as well as within autografts – currently the most reliable clinically-available bridging strategy. However, host Schwann cells generally fail to infiltrate large-gap acellular scaffolds, resulting in markedly inferior outcomes and motivating the development of next-generation bridging strategies capable of fully exploiting the inherent pro-regenerative capability of Schwann cells. We sought to create preformed, implantable Schwann cell-laden microtissue that emulates the anisotropic structure and function of naturally-occurring bands of Büngner. Accordingly, we developed a biofabrication scheme leveraging biomaterial-induced self-assembly of dissociated rat primary Schwann cells into dense, fiber-like three-dimensional bundles of Schwann cells and extracellular matrix within hydrogel micro-columns. This engineered microtissue was found to be biomimetic of morphological and phenotypic features of endogenous bands of Büngner, and also demonstrated 8 and 2× faster rates of axonal extension *in vitro* from primary rat spinal motor neurons and dorsal root ganglion sensory neurons, respectively, compared to 3D matrix-only controls or planar Schwann cells. To our knowledge, this is the first report of accelerated motor axon outgrowth using aligned Schwann cell constructs. For translational considerations, this microtissue was also fabricated using human gingiva-derived Schwann cells as an easily accessible autologous cell source. These results demonstrate the first tissue engineered bands of Büngner (TE-BoBs) comprised of dense three-dimensional bundles of longitudinally aligned Schwann cells that are readily scalable as implantable grafts to accelerate axon regeneration across long segmental nerve defects.

**GRAPHICAL ABSTRACT 1:**
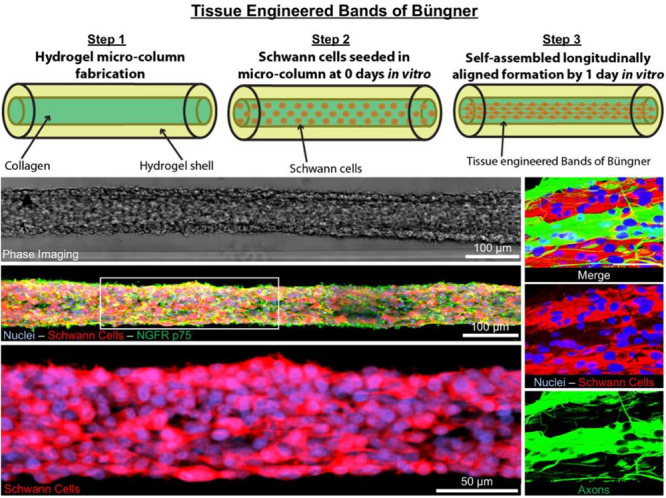
Tissue Engineered Bands of Büngner (TE-BoB) are comprised of longitudinally-aligned Schwann cells within a hydrogel micro-column. By 1 day *in vitro*, the Schwann cells rapidly self-assemble into the longitudinal organization. By 4 days *in vitro*, the Schwann cells form a dense bundle as seen using phase microscopy. High-resolution confocal imaging enabled visualization of longitudinally-aligned Schwann cell processes. Notably, when co-cultured with a neuronal population plated on one end of the construct, axons rapidly accelerated through the TE-BoB construct along the aligned Schwann cells mimicking a key feature found in the naturally-occurring bands of Büngner necessary for regeneration after injury.

## Introduction

Peripheral nerve injury (PNI) presents in 2–5% of all trauma cases, such as sports-related injuries, vehicle accidents, combat situations, or iatrogenic damage (Robinson, 2000; Pfister et al., 2011; Wang et al., 2017). PNIs are often associated with poor functional recovery due to inherently slow axonal regeneration (∼1 mm/day) and prolonged periods of denervation that decrease the capacity for axon regeneration (Ruijs et al., 2005; Gordon et al., 2011). Nerve injuries are primarily classified based on the extent of damage to the overall nerve structure, ranging from a mild crush or stretch injury to a complete disconnection requiring surgical reconstruction to reconnect the proximal and distal nerve stumps (Ruijs et al., 2005; Ali et al., 2014, 2015; Zager, 2014). The most severe nerve injuries are disconnections with a segmental defect that require implantation of grafting material, such as a biological or synthetic nerve conduit, to guide regeneration (Pfister et al., 2011). Poor regeneration is often associated with severe nerve injury, especially with long segmental defects and/or long total regenerative distances.

After nerve injury, axons in the distal nerve segment undergo Wallerian degeneration—the rapid degradation of axons disconnected from the proximal neuronal cell body in or near the spinal cord. Schwann cells distal to the injury dedifferentiate and align with the basal lamina forming highly longitudinally-oriented parallel tubular structures called the bands of Büngner (Salzer, 2015). These pro-regenerative micro-structures serve as a natural living scaffold that facilitates targeted reinnervation of the denervated end-target(s) (Gordon and Stein, 1982; Jessen and Mirsky, 2016).

**FIGURE 1:**
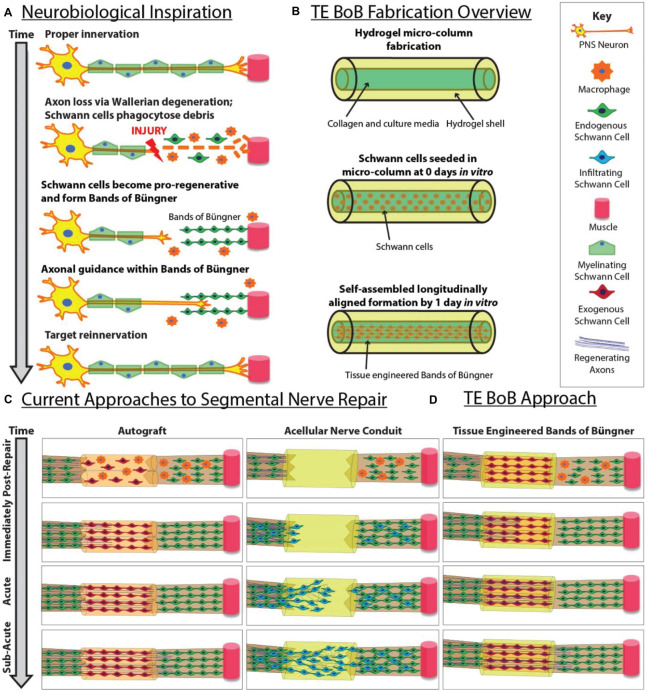
Inspiration, fabrication protocol, and proposed applications for TE-BoBs. This diagram illustrates **(A)** features of endogenous peripheral nerve regeneration that inspired TE-BoB design, **(B)** TE-BoB general fabrication process, **(C)** current techniques to guide injured axons from the proximal nerve to the distal nerve closing the nerve gap, and **(D)** application of the TE-BoB for nerve regeneration and reinnervation.

In cases of segmental nerve defects, grafting is often required to replace the lost nervous tissue with a permissive scaffold that bridges the gap between the nerve stumps (Ray and Mackinnon, 2010). Despite recent advancements in biomaterial development and tissue engineering, autografts remain the most common bridging strategy for long segmental nerve defects. In contrast to alternative commercially-available strategies, such as nerve guidance conduits or acellular nerve allografts, autografts are natural living scaffolds that provide anisotropic structural support as well as neurotrophic support and a myriad of other signaling molecules actively secreted by cells residing in the scaffold (Zhang et al., 2019). Similar to the pro-regenerative response in the distal nerve segment, the Schwann cells found within the donor nerve of the autograft dedifferentiate and form bands of Büngner along the basal lamina (Pfister et al., 2011). Autografts likely promote functional recovery and enable rapid axonal extension across segmental defects by providing endogenous structural support as well as a rich supply of growth factors from the resident Schwann cells (Figure 1).

In contrast, for nerve guidance conduits and acellular nerve grafts, infiltration of host Schwann cells from both nerve stumps is necessary to enable axon re-growth from the proximal stump and across the defect (Kaplan et al., 2015). This process – involving Schwann cell proliferation, migration, and alignment – occurs relatively slowly, likely contributing to reduced rates of axon regeneration across acellular bridging strategies as compared to autografts (Katiyar et al., 2020; Maggiore et al., 2020). In addition, acellular bridging strategies are generally inadequate in enabling axon regeneration across long segmental defects (e.g., >3 cm), which is believed to be due to an inability of host Schwann cells to fully infiltrate the grafts. While not completely understood, this failure may be due to limitations in Schwann cell migratory capacity and/or an insufficient number of proliferative cycles to meet requirements for spanning the graft zone (Saheb-Al-Zamani et al., 2013; Poppler et al., 2016). Moreover, decreased rates and quantity of regenerating axons across acellular grafts also results in prolonged periods of distal nerve and muscle denervation. In these cases, Schwann cells are unable to sustain the bands of Büngner phenotype for prolonged periods without direct axonal contact (Pfister et al., 2011). Thus, prolonged denervation of the distal Schwann cells ultimately results in diminished regenerative capacity and decreased targeted reinnervation (Jessen and Mirsky, 2019). Therefore, greater functional recovery can be achieved by increasing the rate of axon regeneration across a segmental defect to best leverage the regenerative capacity of the bands of Büngner across the full length of the distal nerve segment.

While the use of autografts in peripheral nerve repair surgery most consistently results in positive outcomes, this strategy is far from an ideal solution. Indeed, the procedure of autograft harvest inherently involves the deliberate creation of an additional functional nerve deficit, as well as having limited donor nerve availability for long gap nerve repair and/or polytrauma, and often presenting diameter mismatch at the interface between the injured nerve and the donor nerve. As an alternative, the development of a tissue engineered living scaffold containing Schwann cells may recapitulate pro-regenerative architecture and accelerate host axon regeneration (Figure 1). Various approaches have been pursued to increase Schwann cell alignment and enhance neurite extension *in vitro* (Bozkurt et al., 2007, 2009). For use *in vivo*, the fabrication of cell-laden nerve guidance conduits is intended to induce Schwann cell organization into the bands of Büngner *in situ* and subsequently enable rapid axonal regeneration (Das et al., 2015, 2017). Indeed, transplantation of Schwann cells, mesenchymal stem cells, or Schwann cell-like cells encased in hydrogel matrices have been investigated as a potential therapeutic strategy for more challenging nerve repair; however, previous approaches have yet to directly recreate the intra- and inter-cellular morphology and phenotype of the bands of Büngner prior to implant (Daud et al., 2012; Georgiou et al., 2013; Weightman et al., 2014; Kornfeld et al., 2016).

We have previously utilized microtissue engineering strategies to develop various living scaffolds as advanced approaches for regenerative medicine (Struzyna et al., 2015, 2017; Winter et al., 2016; Katiyar et al., 2018; O’Donnell et al., 2018). Here, we report the development of the first miniaturized, transplantable, preformed tissue engineered bands of Büngner (TE-BoB). Specifically, we demonstrate the facile biofabrication of TE-BoBs exploiting principles of material-guided cell self-assembly, as well as characterization of the resulting cellular structure, phenotype, and functional capacity to accelerate motor and sensory axonal outgrowth *in vitro*. TE-BoBs are comprised of self-assembled longitudinally-aligned Schwann cells that can facilitate axon outgrowth and bundling *in vitro.* Remarkably, we found that TE-BoBs achieved motor axon and sensory axon growth rates that were at least 10.7 and 4.3× faster, respectively, than rates achieved by alternative previously published Schwann cell-mediated strategies (Phillips et al., 2005; Gingras et al., 2008; Daud et al., 2012; Georgiou et al., 2013; Hyung et al., 2015). For translational consideration, we demonstrate proof-of-concept of TE-BoB fabrication using human gingiva stem cell-derived Schwann cells. TE-BoBs are a novel living scaffold suitable for use in follow-on studies to assess their ability to accelerate axon regeneration across segmental defects in an *in vivo* model of PNI.

## Materials and Methods

All procedures were approved by the Institutional Animal Care and Use Committees at the University of Pennsylvania and the Michael J. Crescenz Veterans Affairs Medical Center and adhered to the guidelines set forth in the NIH Public Health Service Policy on Humane Care and Use of Laboratory Animals (2015).

### Hydrogel Micro-Column Fabrication

Three-dimensional hollow hydrogel micro-columns were formed to promote alignment and bundling of Schwann cells throughout the lumen. This protocol was adapted from our previous studies utilizing a similar microtissue engineering technique to align astrocytes for a tissue engineered rostral migratory stream (Winter et al., 2016; Katiyar et al., 2018; O’Donnell et al., 2018). All hollow micro-columns were fabricated with an inner diameter (ID) of 300 μm, an outer diameter (OD) of 701 μm, a length of 5 mm, and an agarose concentration of 3%. Agarose is a biocompatible, optically transparent, and relatively inert biomaterial that lacks adhesive ligands, which allows for specific investigation of the relationship between the cells and the collagen extracellular matrix (ECM) coating the inner lumen. Approximately 2.0 μL of collagen (1 mg/ml) was microinjected into each lumen. A polymerization/dehydration time of 3 h allowed collagen to coat the inner lumen of the micro-columns, creating an outer agarose shell, inner collagen coating, and hollow core. Corresponding 2D controls were prepared in 10-mm petri dishes, pretreated with poly-L-lysine overnight, and then rinsed three times. Approximately 2.0 μL of collagen (1 mg/ml) was added to the center of the dish. The 2D controls were returned to the incubator to polymerize for 3 h similar to the micro-columns.

### Primary Schwann Cell Culture

Primary Schwann cells were obtained from the Salzer Lab (NYU) and subsequently passaged every 7 days during the duration of this study. Schwann cells were cultured in minimum essential media (Thermo Fisher Scientific, Gibco 11095072), 10% fetal bovine serum (FBS), 10 ng/mL recombinant human neuregulin-1-β1 EGF domain (R&D, 396-HB-050), 2.5 μM forskolin (Sigma, F-6886), and 1% Penicillin/Streptomycin (Kim and Maurel, 2009). The resulting cell suspension was split to maintain the cell line and to seed the constructs. Collagen-coated micro-columns were seeded with approximately 2 μL of cell suspension (1.1 × 10^5^–1.3 × 10^7^ cells/mL). Additional cell suspension was plated onto 2D polymerized collagen with identical cell suspension concentration and volume. For TE-BoB fabrication and 2D controls, Schwann cells were incubated for 30 min to allow for adhesion before carefully submerging them in 2 mL Schwann cell growth medium. A total number of *n* = 35 TE-BoBs and control cultures were generated for this study.

### Primary Dorsal Root Ganglion (DRG) and Spinal Motor Neuron (MN) Isolation and Co-culture

Dorsal root ganglion (DRG) explants and spinal cords were isolated from embryonic day 16 Sprague-Dawley rats (Charles River, Wilmington, MA, United States). DRG explants were stored overnight in Hibernate-E. MN aggregates were formed from dissociated spinal MNs isolated from embryonic spinal cords using an Optiprep density gradient and subsequent forced-aggregation as previously described (Katiyar et al., 2019). Briefly, dissociated MNs were plated in a “reverse pyramid” well comprised of polydimethyl siloxane. Each well received 12 μL of 100,000 dissociated MNs, and centrifuged at 1500 RPM for 5 min. Motor neuron aggregates were incubated overnight in media.

At 1 day *in vitro*, MN aggregates or DRG explants comprised of sensory neurons (SNs) were plated under stereoscopic magnification using fine forceps on one end of a TE-BoB or acellular collagen-coated micro-column, or on top of Schwann cells seeded on planar collagen. Cultures were allowed to adhere at 37°C and 5% CO_2_.

Media was changed on the next day and then every other day. For these co-culture studies, the media was Neurobasal media and 10% FBS first conditioned in a flask of astrocytes overnight, and then supplemented the next day with 37 ng/mL hydrocortisone, 2.2 μg/mL isobutylmethylxanthine, 10 ng/mL brain-derived neurotrophic factor, 10 ng/mL ciliary neurotrophic factor, 10 ng/mL cardiotrophin-1, 10 ng/mL glial cell line-derived neurotrophic factor, 2% B-27, 20 ng/mL nerve growth factor, 4 μM uridine, 4 μM 5-FdU, 2 mM L-glutamine, 417 ng/mL forskolin, 1 mM sodium pyruvate, 0.1 mM β-mercaptoethanol, 2.5 g/L glucose, and 10 ng/ml recombinant human neuregulin-1-β1 epidermal growth factor domain (Katiyar et al., 2019).

### Immunocytochemistry

All samples were fixed at 4 days *in vitro*. Immunocytochemistry was completed to evaluate Schwann cell and neuronal phenotype, assess the presence of collagen, and characterize the cytoarchitecture within the micro-column and 2D cultures. Briefly, cultures were fixed in 4% formaldehyde for 30 min, washed in phosphate buffered saline (PBS), and permeabilized in 0.3% Triton X100 plus 4% normal horse serum (NHS) for 1 h. Cultures were incubated with primary antibodies in PBS + 4% serum solution) at 4°C for 12 h. To label Schwann cells, guinea pig anti-S100β (Synaptic Systems 287004; 1:500; intracellular calcium-binding protein) and rabbit anti-p-75 (Sigma N3908; 1:500; nerve growth factor receptor) were used. To evaluate neurite outgrowth, cultures were stained with mouse anti-beta tubulin III (Tuj1) (Sigma T8578; 1:500) to label all axons and neurons. To assess the distribution of collagen ECM, rabbit anti-collagen I (Abcam ab34710; 1:500) was used. After rinsing, appropriate secondary antibodies (1:500 in PBS + 4% NHS; anti-mouse 488, Invitrogen, A21202; anti-rabbit 488, Life Technology, A21206; anti-guinea pig 568, Sigma SAD4600038; and/or anti-rabbit 647, Invitrogen, A31573) were applied at room temperature for 2 h and Hoechst (Invitrogen H3570; 1:10,000) was then added to label all nuclei.

### Microscopy and Data Acquisition

Schwann cell cultures and constructs were imaged using phase contrast microscopy at 1 and 4 days *in vitro* with a Nikon Inverted Eclipse Ti-S microscope with digital image acquisition using a QiClick camera interfaced with Nikon Elements Basic Research software (4.10.01). Confocal images were taken at 4 days *in vitro* using a Nikon A1RSI laser scanning confocal microscope.

All images acquired for comparative analyses were captured with identical acquisition settings. Samples were fluorescently imaged using a Nikon A1Rsi Laser Scanning Confocal microscope with a 10× (CFI Plan Apo Lambda 10×; n.a. 0.45) or 16x objective (CFI75 LWD 16× W; n.a. 0.8).

Image post-processing and quantification was completed using FIJI (Fiji Is Just ImageJ) software platform (Schindelin et al., 2012). Nikon image files were imported into FIJI via the Bioformats function and each channel was split into individual channels. To minimize potential bias, trained researchers were given only the axonal channel containing a randomly-coded ID. Images were rotated to align the horizontal axis with the inner lumen. Schwann cell orientation was measured relative to the horizontal axis from 150 individual S100+ cells evenly distributed across TE-BoBs at 4 days *in vitro*. Neurite length was quantified by measuring the distance from the edge of the neuronal bodies. Neurite directionality was analyzed qualitatively.

To compare the degree of axon fasciculation, a macro for automated image processing analyses was designed to minimize any potential bias. Background subtraction was applied to all images using the rolling ball method with a diameter of 100 pixels (O’Donnell et al., 2016). Images were rotated to ensure constructs were parallel to the horizontal axis. A 5,000 μm × 300 μm (length × width) region of interest (ROI) was placed starting from the edge of the neuronal-axonal interface. Axonal segments were isolated from the Tuj1 channel using MaxEntropy thresholding and subsequently quantified using the “Analyze Particles” function on features with an area greater than 10 μm^2^ to minimize noisy particles and circularity between 0 and 0.3 to eliminate circular artifacts. The total bundle area of the segmented regions, average size of each bundle, and area percent covered were calculated per construct. Mean values were obtained by averaging across constructs for further statistical analyses.

To compare neuronal density within the aggregate region, Tuj1 expression was measured using an automated image processing macro to minimize any potential bias. The neuron region was isolated from the axonal region by placing a 500 μm × 300 μm (length × width) in a representative area in the middle of the neuronal population. Three representative ROIs (100 μm × 100 μm) were selected for further analysis. Background subtraction was applied to all images using the rolling ball method with a diameter of 50 pixels, which appeared to remove smaller and more diffuse axonal staining (O’Donnell et al., 2016). Neurons were isolated from the Tuj1 channel using MaxEntropy thresholding and subsequently quantified using the “Analyze Particles” function on features with an area greater than 10 μm^2^ to minimize noisy signal. The percent area covered was calculated for each ROI and averaged per construct. Therefore, in this study, the percentage of the Tuj1 expression within the aggregate may be considered as a surrogate marker for neuronal health. Mean values were obtained by averaging across constructs for further statistical analyses.

### Study Design and Statistical Analysis

Initial TE-BoB characterization was completed using phase imaging and immunocytochemistry (*n* = 7). Various conditions were studied to quantify the effects of aligned Schwann cell bundles on sensory and motor axon outgrowth *in vitro*. The independent variables included Schwann cell configuration (2D culture vs. 3D bundling) and aligned Schwann cell presence (Schwann cell/collagen vs. collagen-only constructs), while the dependent variables included neurite length and directionality. These variables were selected to assess the regenerative promotion and directional guidance provided by TE-BoBs.

Experimental groups included hydrogel micro-column constructs with bundles of collagen and aligned Schwann cells (TE-BoBs), plated with either one SN explant (*n* = 4) or one MN aggregate (*n* = 6). The 3D control groups contained collagen-coated hydrogel micro-columns plated with one SN explant (*n* = 5) or one MN aggregate (*n* = 5) in the absence of Schwann cells. The 2D control groups consisted of Schwann cell cultures on a planar bed of collagen, each plated with either one SN explant (*n* = 3) or one MN aggregate (*n* = 5).

At 4 days *in vitro*, the length of neurite outgrowth was measured linearly from the nearest soma aggregate edge to the axon terminal of the longest neurite. Neurite outgrowth was measured for each culture from confocal z-stack maximum projections and analyzed using FIJI software (Schindelin et al., 2012). Mean neurite length was determined for each group and statistically analyzed using one-way ANOVA followed by Tukey’s multiple comparison test to determine statistical significance (*p* < 0.05 required for significance). For the axon fasciculation assay, mean values were compared between TE-BoBs and constructs lacking Schwann cells by two-tailed unpaired Student’s *t*-tests (α = 0.05). Values are reported as mean ± SEM, unless otherwise noted. Statistical testing was performed in GraphPad Prism 8 for Windows 64 bit.

### Human Gingiva-Derived TE-BoB Fabrication

In a separate proof-of-concept experiment, TE-BoBs were fabricated using Schwann cell-like cells induced from human gingiva-derived mesenchymal stem cell (GMSC) source using a previously established derivation protocol (Zhang et al., 2018a,b). Human gingival tissues were obtained as remnants of discarded tissues from healthy human subjects aged from 20 to 40 years old, who underwent routine dental procedures. Informed consents were obtained from all subjects and all procedures were performed under the approved Institutional Review Board (IRB) protocol at University of Pennsylvania. Primary GMSCs were cultured and maintained in complete alpha-minimum essential medium (α-MEM) supplemented with 1% L-glutamine, 10% FBS (Zen Bio) and 1% penicillin/streptomycin at 37°C with 5% CO_2_ as describe previously (Zhang et al., 2009). Cells less than 8 passages were used for experiments.

For induction of GMSC-derived neural crest stem-like cells (NCSCs) (Zhang et al., 2018b), GMSCs were plated in poly-L-ornithine pre-coated culture dishes and cultured in media consisting of 50% DMEM/F12 (Life Technologies, 11330-032) and 50% Neurobasal medium (Life Technologies, 21103-049) supplemented with 20 ng/mL human basic fibroblast growth factor (PeproTech, 100-18C), 20 ng/mL human epidermal growth factor (PeproTech, AF-100-15), 55 μM β-mercaptomethanol (Life Technologies, 21985-023), 1% N2 (Life Technologies, 17-502-048), 1% B27 (Life Technologies, 17-502-044), and 100 units penicillin, 100 μg/mL streptomycin (Life Technologies, 15140-122). Six days later, cells were harvested for Schwann cell induction (Zhang et al., 2018a,b). Briefly, GMSC-derived NCSCs were cultured Schwann cell differentiation media consisting of α-minimal essential media (Life Technologies, 12561-056) supplemented with 10% fetal bovine serum (Zenbio Inc., SER-500), 35 ng/mL all *trans*-retinoic acid (Sigma, R2625), 5 μM forskolin (Cayman Chemical, 11018), 10 ng/mL human basic fibroblast growth factor (PeproTech, 100-18C), 5 ng/mL platelet-derived growth factor-AA (PeproTech, 100-13A), 200 ng/mL β-heregulin (PeproTech, 100-03), 100 units penicillin, 100 μg/mL streptomycin (Life Technologies, 15140-122).

Following induction for 7 days, GMSC-derived Schwann cell-like cells were dissociated and plated in micro-columns as described above (2.5 × 10^5^–3.0 × 10^6^ cells/mL). Immunocytochemistry was performed on planar cultures at 3 days *in vitro* to label for nuclei (DAPI) and Schwann cells (S100β) as described above. Phase microscopy was performed at 3 days after TE-BoB fabrication.

## Results

### Schwann Cell Seeding, Process Extension, and Bundling

**FIGURE 2:**
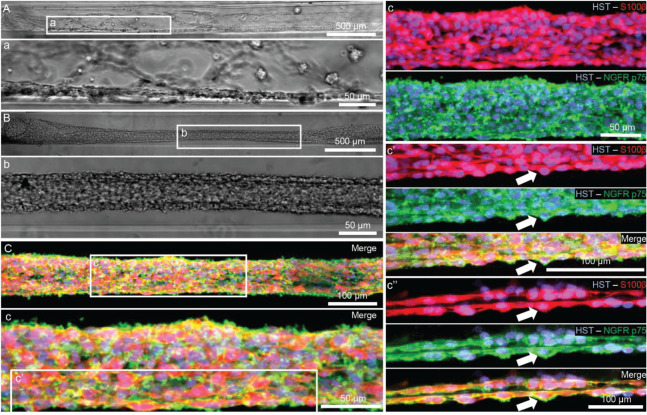
Characterization of Schwann cell constructs following self-assembly fabrication process using phase-imaging, immunocytochemistry, and confocal microscopy. **(A,B)** Phase microscopy was utilized to visualize Schwann cells seeded in a 300 μm ID agarose hydrogel micro-column. **(A)** At 1 day *in vitro*, Schwann cells were found adhering to the collagen ECM coating the inner lumen of the micro-column. **(a)** Higher magnification revealed the cells begun to self-assemble into cables and exhibit process-bearing morphology. **(B)** By 4 days *in vitro*, the Schwann cells formed dense bundles within the inner lumen of the agarose micro-column. **(b)** These bundles appeared highly organized comprised of Schwann cells with longitudinally-aligned processes. **(C)** Morphometric assessment of the dense bundles at 4 days *in vitro* revealed Hoechst (HST)-positive cells with elevated expression of S100b and NGFR p75, common Schwann cell markers. **(c)** Under higher magnification, most of the Schwann cells co-localized with NGFR p75R and exhibited bipolar morphologies with elongated processes, a common phenotype during nervous system development and regeneration. **(c′)** Max projection image showing a high density of aligned Schwann cells denoted by arrows. **(c′′)** Single z-plane frame from the same image illustrating NGFR p75 expression on the membrane of highly aligned S100β positive Schwann cells denoted by arrows. Scale bars: **(A,B)** 500 μm, **(a,b)** 50 μm, **(C)** 100 μm, **(c)** 50 μm, **(c″)** 100 μm.

To biofabricate TE-BoBs, Schwann cells were seeded in an agarose hydrogel micro-column 5 mm long with OD of 701 μm, ID of 300 μm, and collagen-coated inner lumen. By 1 day *in vitro*, Schwann cells that were seeded in the agarose micro-columns had adhered to the collagen ECM coating the inner lumen, began to exhibit a process-bearing morphology, and eventually self-assembled into a dense network along the inner lumen of the micro-column (Figure 2A). By 4 days *in vitro*, as the Schwann cells continued to remodel the collagen ECM, the density of the Schwann cells rapidly increased, forming a singular dense bundle in the lumen several millimeters long and, in most cases, spanning the entire 5 mm lumen of the micro-column (Figure 2B). At 4 days *in vitro*, the bundled Schwann cells exhibiting a bipolar morphology aligned along the lumen of the micro-column (−1.2° ± 10.1° relative to longitudinal axis; mean ± standard deviation) and demonstrated consistent co-expression of both S100β and nerve growth factor receptor (NGFR p75) (Figure 2C).

### Longitudinally-Aligned Schwann Cells Accelerate Neurite Outgrowth

**FIGURE 3:**
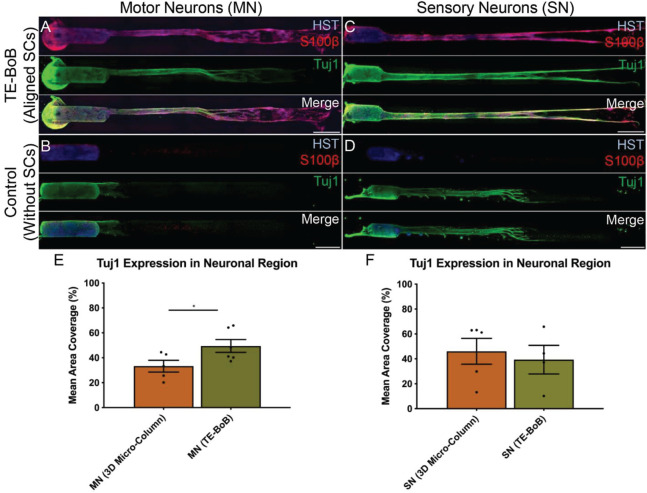
Comparison of three-dimensional neurite outgrowth in TE-BoBs and collagen-only constructs. A neurite outgrowth assay was performed to elucidate the effect of longitudinally aligned Schwann cells (SCs) on axonal extension by carefully plating a population of motor neurons **(A,B)** or sensory neurons **(C,D)** on one end of a TE-BoB containing aligned Schwann cells or collagen-only micro-column at 1 day *in vitro* following fabrication. Constructs were fixed at 4 days *in vitro* and stained for nuclei (HST), Schwann cells (S100β) and axons (Tuj1). Highly aligned motor and sensory neurites were visualized extending from the neurons and spanning the Schwann cell constructs **(A,C)**. Axon outgrowth appeared more disorganized in collagen control constructs lacking Schwann cells **(B,D)**. In contrast, axonal outgrowth within the cellular constructs appeared to closely follow the dense bundles of longitudinally-aligned Schwann cells. **(E)** Detailed image analysis of the neuronal population revealed greater Tuj1 expression in motor neurons co-cultured with Schwann cells compared to motor neurons cultures lacking Schwann cells. **(F)** No significant differences in Tuj1 expression were found in sensory neurons with or without co-cultured Schwann cells. Scale bar: 500 μm. ^∗^*p* < 0.05.

**FIGURE 4:**
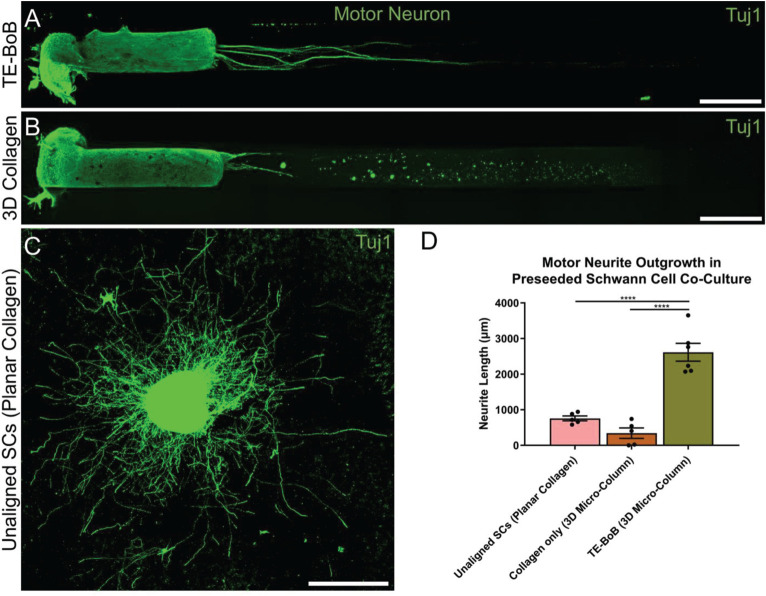
Quantification of motor neuron outgrowth in TE-BoBs. Neurite outgrowth was measured at 4 days *in vitro* after plating a motor neuronal aggregate in a micro-column containing aligned Schwann cells **(A)** or collagen only control **(B)**, or on a bed of Schwann cells in 2D **(C)**. **(D)** Greater neurite outgrowth was found in TE-BoBs (i.e., aligned Schwann cells in a 3D micro-column) compared to the collagen only control and the 2D planar Schwann cell control. Error bars represent standard error of mean. Scale bar: 500 μm. ****p < 0.0001.

**FIGURE 5:**
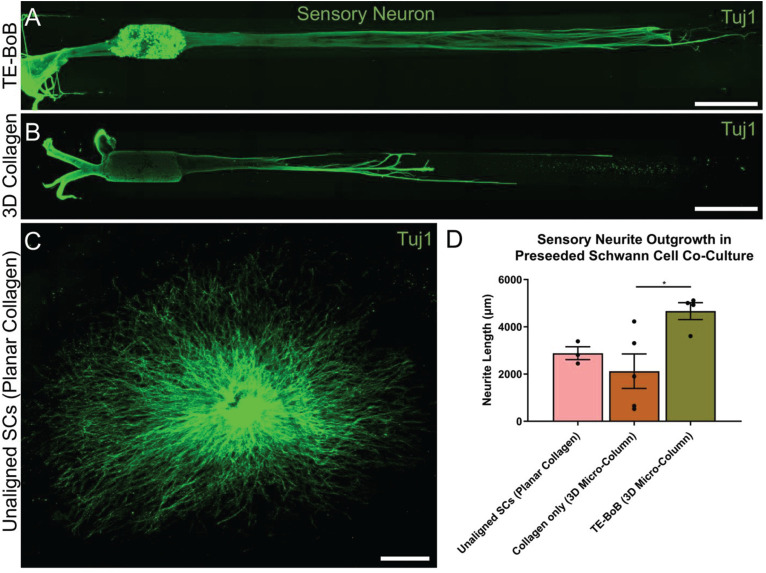
Quantification of sensory neuron outgrowth in TE-BoBs. Neurite outgrowth was measured at 4 days *in vitro* after plating sensory neurons (DRG explant) in a micro-column containing aligned Schwann cells **(A)** or collagen only control **(B)**, or on a bed of Schwann cells in 2D **(C)**. **(D)** Greater neurite outgrowth was found in TE-BoBs (i.e., aligned Schwann cells in a 3D micro-column) compared to the collagen only control. Error bars represent standard error of mean. Scale bar: 500 μm. *p < 0.05.

To evaluate the effect of TE-BoBs on motor and sensory neurite outgrowth, we compared axonal extension within TE-BoB micro-columns containing aligned Schwann cells to 3D control micro-columns containing only collagen and 2D controls containing Schwann cells on collagen in planar culture. Here, we added either MN or SN aggregates to one end of micro-columns at 1 day *in vitro*, which were then returned to culture until 4 days *in vitro* (Figures 3A–D). Although there was some S100β positivity within the MN and SN aggregates and at the interface with Tuj1 positive axons, the absence of S100β positivity within the collagen micro-columns lacking Schwann cells suggested that there was no Schwann cells migration into the micro-column. In this study, Tuj1 expression within the aggregate region was measured to provide an indirect measurement of neuron density and serve as a surrogate marker for neuronal health. Greater area of Tuj1 expression was found in MN aggregates containing aligned Schwann cells (mean: 49.5% ± 12.6%; range: 37.2 – 65.8%; *n* = 6) compared to the control collagen micro-column (*p* < 0.05; mean: 33.3% ± 10.5%; range: 20.2–44.5%; *n* = 5) (Figure 3E). No significant differences were found between SNs co-cultured with aligned Schwann cells (mean: 39.4% ± 22.9%; range: 10.2–65.8%; *n* = 4) and the control collagen micro-column (mean: 46.1% ± 23.2%; range: 13.2–63.2%; *n* = 5) (Figure 3F). The presence of longitudinally-aligned Schwann cells resulted in the longest axonal outgrowth for both sensory and motor neurite assays. Increased neurite outgrowth was observed in TE-BoBs containing a MN aggregate (mean: 2614.6 μm ± 249.9; range: 2093.6–3652.8 μm; *n* = 6) compared to control collagen micro-columns (*p* < 0.0001; mean: 341.8 μm ± 145.7 μm; range: 0–742.3 μm; *n* = 5), and 2D Schwann cell co-culture (*p* < 0.0001; mean: 756.5 μm ± 67.4 μm; range: 582.5–950.0 μm; *n* = 5) (Figure 4). Similarly, greater sensory axon outgrowth was observed in TE-BoBs with a SN explant (mean: 4665.1 μm ± 355.4; range: 3605.2–5118.3 μm; *n* = 4) compared to control collagen columns (*p* < 0.05; mean: 2122.2 μm ± 728.1 μm; range: 522.9–4226.1 μm; *n* = 5). No significant difference was found compared to SN explants plated on 2D Schwann cell control cultures (mean: 2883.1 μm ± 272.3 μm; range: 2449.6–3385.4 μm; *n* = 3) (Figure 5).

### TE-BoBs Enhance Axon Area and Fasciculation

Detailed image analysis was performed on TE-BoBs and constructs lacking Schwann cells using automated image analysis across a 5000 μm × 300 μm ROI. Percent axon area coverage, total bundle area, average bundle size, and average bundle count were analyzed.

**FIGURE 6:**
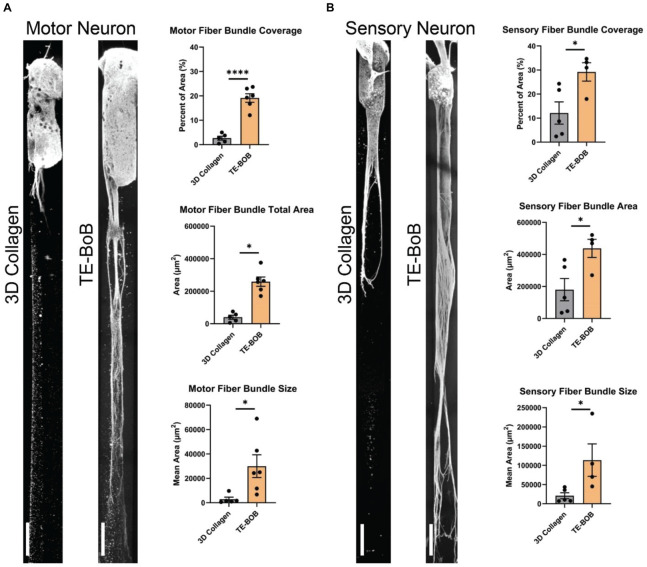
Assessment of motor and sensory axon bundling in TE-BoBs. Axon bundling was measured at 4 days *in vitro* after plating either a **(A)** spinal cord motor neuron aggregate or **(B)** DRG explant containing sensory neurons on the end of a 3D micro-column comprised of 3D collagen ECM (control) or aligned Schwann cells (TE-BoB). Fluorescent images were gray-scaled and a 5,000 μm × 300 μm ROI was drawn starting at the edge of the neuronal-axonal interface. An automated macro was applied over each ROI to identify regions of bundled axons after excluding small debris and/or artifact. The presence of longitudinally-aligned Schwann cells resulted in the formation of large axon bundles covering the lumen of the micro-column. Error bars represent standard error of mean. Scale bar: 250 μm. ^∗^*p* < 0.05, ^****^p < 0.0001.

The presence of longitudinally-aligned Schwann cells in TE-BoBs resulted in large MN bundles within the lumen compared to control constructs lacking Schwann cells (Figure 6A). The percent area covered by MN bundles in TE-BoBs (mean: 19.1% ± 1.7%; range: 12.1–23.7%) was greater than the control constructs lacking Schwann cells (*p* < 0.0001; mean: 2.7% ± 0.8%; range: 0.4–5.0%). The total area covered by MN bundles in TE-BoBs (mean: 258,947 μm^2^ ± 28,318 μm^2^; range: 170,591–375,707 μm^2^) was greater than control constructs lacking Schwann cells (*p* < 0.001; mean: 40,042 μm^2^ ± 12,506 μm^2^; range: 5778–75,356 μm^2^). The average size of the MN bundles in TE-BoBs (mean: 29,939 μm^2^ ± 9328 μm^2^; range: 6768 μm^2^–62,185 μm^2^) was greater than control constructs lacking Schwann cells (*p* < 0.05; mean: 2933 μm^2^ ± 1708 μm^2^; range: 577.8–9131 μm^2^). Therefore, dense motor axonal bundles were formed in the presence of longitudinally-aligned Schwann cells compared to small bundles with less dense axons within control micro-columns containing only collagen.

Similar to these findings with MNs, the presence of longitudinally-aligned Schwann cells in TE-BoBs also resulted in large SN bundles within the lumen compared to control constructs lacking Schwann cells (Figure 6B). The percent area covered by SN bundles in TE-BoBs (mean: 29.2% ± 3.8%; range: 17.8–34.7%) was greater than the control constructs lacking Schwann cells (*p* < 0.05; mean: 12.1% ± 4.6%; range: 2.4–24.3%). The total area covered by SN bundles in TE-BoBs (mean: 437,991 μm^2^ ± 56,875 μm^2^; range: 270,358 μm^2^–521,157 μm^2^) was greater than control constructs lacking Schwann cells (*p* < 0.05; mean: 179,987 μm^2^ ± 69,323 μm^2^; range: 35,625–366,309 μm^2^). The average size of the SN bundles in TE-BoBs (mean: 113,539 μm^2^ ± 42,174 μm^2^; range: 45,060 μm^2^–234,720 μm^2^) was greater than control constructs lacking Schwann cells (*p* < 0.05; mean: 20,890 μm^2^ ± 7761 μm^2^; range: 7125–43,473 μm^2^). Overall, the presence aligned Schwann cells resulted in the formation of dense sensory axonal bundles, as compared to small bundles with more diffuse axons within control micro-columns containing only collagen.

### Axon-Schwann Cell Interactions Within TE-BoBs Mimic Natural Bands of Büngner and Provide Longitudinal Directionality

**FIGURE 7:**
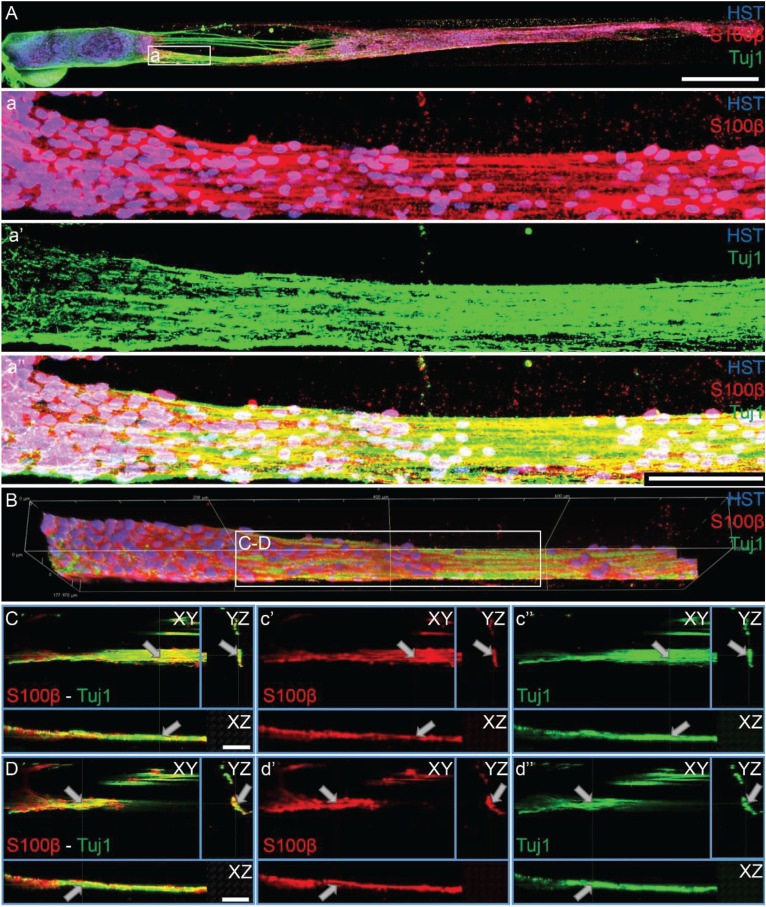
Motor axons extending through longitudinally-aligned Schwann cells in TE-BoBs. **(A)** Motor axons extending from the neuronal aggregate (Tuj1) appear to interact closely with the highly aligned Schwann cells with bipolar morphology (S100β). **(a)** At higher magnification, highly bundled axons were visualized extending parallel to the longitudinally-aligned Schwann cells. **(B)** Volumetric reconstruction of high resolution confocal z-stack images demonstrating the relationship between the motor axons and longitudinally-aligned Schwann cells in the TE-BoB construct, which resembles the arrangement found between axons and Schwann cells in the pro-regenerative bands of Büngner *in vivo*. **(C,D)** Individual z-planes and orthogonal perspective views from **(B)** are shown to highlight that the axons are extending parallel to the longitudinally-aligned Schwann cells. Arrows denote same area across different perspectives further illustrating the relationship between the motor axons and Schwann cells within the TE-BoB. Scale bars: **(A)** 500 μm, **(a)** 100 μm, **(C,D)** 50 μm.

**FIGURE 8:**
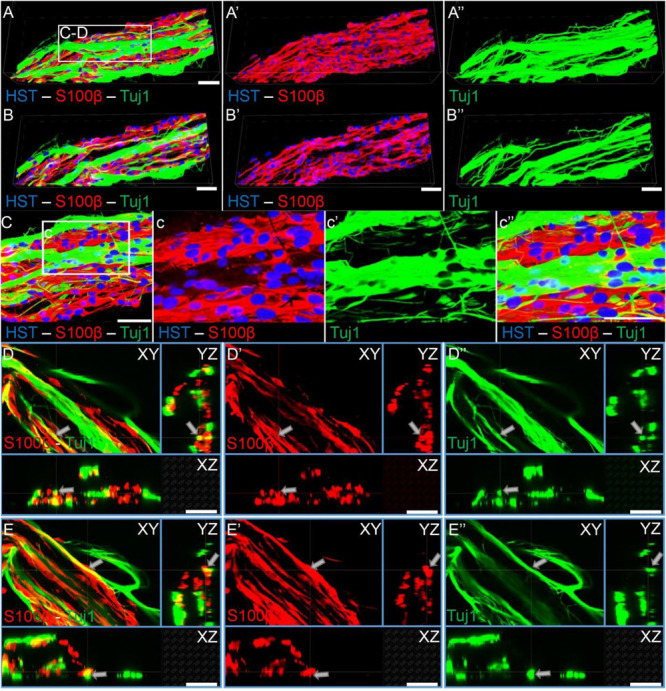
Sensory axons extending through longitudinally-aligned Schwann cells in TE-BoBs. **(A)** Volumetric reconstruction of high resolution confocal z-stack images revealing the close interaction between the sensory axons extending from the DRG explant (Tuj1) and the longitudinally-aligned Schwann cells (S100β). **(B)** By examining specific z-planes within the volumetric reconstruction, axons can be clearly visualized closely following Schwann cells in a columnar organization with bipolar morphology. **(C)** Higher magnification reveals the relationship between the axons and the aligned Schwann cells resembling the arrangement found *in vivo* between axons and Schwann cells within the pro-regenerative bands of Büngner **(D,E)** Individual z-planes and orthogonal perspective views from **(C)** are shown to highlight that the axons are extending parallel to the longitudinally-aligned Schwann cells. Arrows denote same area across different perspectives further illustrating the relationship between the sensory axons and Schwann cells within the TE-BoB. Scale bars: Scale bars: **(A–E)** 50 μm.

As TE-BoBs present longitudinally-aligned Schwann cells in a tight, bundled formation, we also ascertained the structural relationships and directivity of axonal outgrowth on these structures in comparison to growth within 3D micro-columns alone and on 2D control cultures. We found that axonal outgrowth from both MNs and SNs were in direct contact and longitudinally-aligned with the Schwann cell bundles comprising the TE-BoBs (see Figures 4, 5). Axonal extension from the MNs and SNs in the 3D micro-columns primarily occurred within the collagen ECM and was not as bundled as that in the TE-BoBs, although outgrowth was physically constrained by the inner walls of the micro-column. In contrast to these cases, motor and sensory axons extended from the 2D control populations in all directions. At a finer level, axonal outgrowth had a “frayed” appearance in the case of growth within acellular micro-columns as compared to tighter, directed outgrowth along the aligned Schwann cells in TE-BoBs (Figure 6). This frayed outgrowth pattern may be due to axonal growth cones “searching” for guiding signals in acellular constructs, as compared to precisely presented longitudinal cues presented by the bundled Schwann cells in TE-BoBs. Building on this observation, high resolution confocal imaging further revealed a familiar spatial relationship between the growing axons and longitudinally-aligned Schwann cells. Axons extending from both MN aggregates (Figure 7) and SN explants (Figure 8) grew along and through these dense bands of aligned Schwann cells comprising the TE-BoBs in a manner reminiscent of *in vivo* axon regeneration within bands of Büngner.

### Human GMSC-Derived Schwann Cell-Like Cells Self-Assemble Into Longitudinally-Aligned Morphology Within TE-BoBs

**FIGURE 9:**
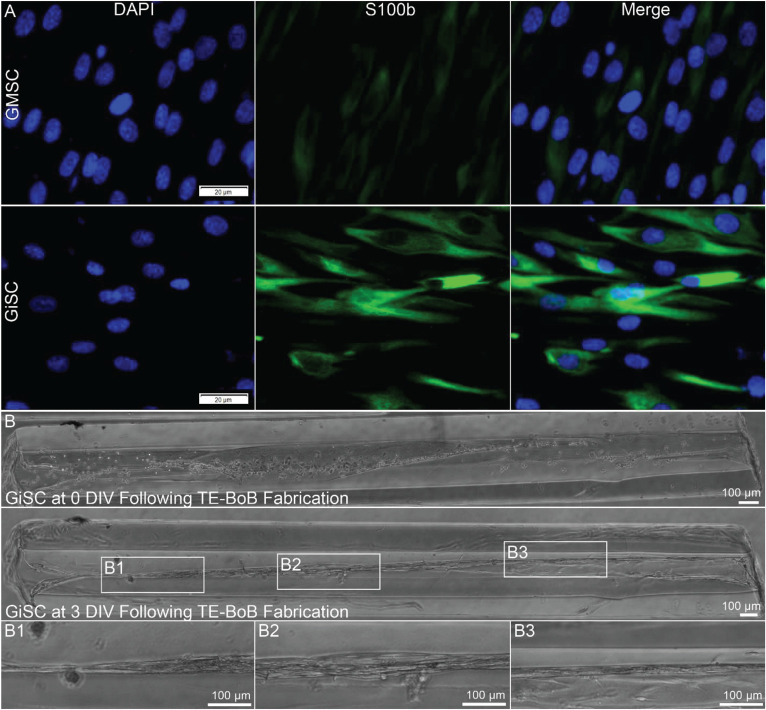
Proof-of-concept fabrication of human gingiva-derived TE-BoBs. In a proof-of-concept demonstration using a potential clinical cell source, TE-BoBs were fabricated using human gingiva-derived mesenchymal stem cells (GMSC) that were induced into a Schwann cell-like phenotype (GiSC). **(A)** Immunocytochemistry was performed on planar cultures at 3 days *in vitro* to label for nuclei (DAPI) and Schwann cells (S100β). Greater S100β expression was found in the GiSC culture, indicating the protein commonly found in Schwann cells was upregulated and expressed in these cells. **(B)** GiSCs were seeded in micro-columns to fabricate human gingiva-derived TE-BoBs and were imaged with phase microscopy over time. At 3 days *in vitro* post TE-BoB fabrication, the cells self-assembled into a longitudinally aligned formation, similar to the rodent derived TE-BoBs. **(B1–B3)** Higher magnification imaging revealed tight bundles of cells with a bipolar morphology within the collagen matrix.

Human Schwann cell-like cells were induced from human GMSC-derive neural crest stem-like cells (Zhang et al., 2018a,b). Prior to TE-BoB fabrication, immunocytochemistry characterization was performed in planar cultures at 3 days *in vitro*. Greater S100β expression was found in human GMSC-derived Schwann cell-like cells compared to the undifferentiated GMSC control culture. The presence of S100β within GMSC-derived Schwann cell-like cell planar culture indicated that these cells expressed a protein commonly found in Schwann cells similar to previous studies (Zhang et al., 2018a,b). Therefore, TE-BoBs were fabricated using these Schwann cell-like cells. By 3 days *in vitro* following fabrication, GMSC-derived Schwann cell-like cells within the TE-BoB self-assembled into a tightly bundled formation that resembled the rodent TE-BoB constructs (Figure 9). These findings demonstrate that the self-assembly mechanisms described for rat Schwann cells are conserved in human Schwann cells, and bode well for the potential of fabricating large-scale human TE-BoBs for future efficacy testing.

## Discussion

Following nerve injury, Schwann cells form bands of Büngner that provide axonal guidance to distal targets for functional reinnervation. To date, autografts remain the gold standard for challenging clinical scenarios, serving as naturally-occurring living scaffolds that facilitate regeneration by providing a permissive substrate with anisotropic structural and neurotrophic support due to indwelling cells. Indeed, resident Schwann cells found in the donor nerve undergo similar phenotypic alterations as denervated Schwann cells found in the distal nerve, and facilitate regeneration by enabling the rapid growth across the defect (Pfister et al., 2011). However, over time, prolonged denervation results in the degradation of the pro-regenerative bands of Büngner, leading to diminished regenerative capacity.

Alternative bridging strategies are generally acellular (i.e., non-living), such as the use of decellularized nerve allografts or biological/synthetic conduits, and unable to consistently support axonal regeneration across defects greater than the critical length of 3 cm (Kornfeld et al., 2019). This is likely due to slow axon regeneration across the defect—which is reliant upon host Schwann cell infiltration and organization across the entire length of the graft region—resulting in prolonged denervation of the Schwann cells in the distal nerve as well as the motor end targets. Indeed, a major challenge for axon regeneration following long gap nerve repair using acellular strategies has been suggested to be senescence of host Schwann cells needed to fill the graft, whereby endogenous Schwann cells lack sufficient proliferative capacity to create enough progeny to fill graft zones more than a few centimeters. Several studies have shown that the expression of senescence markers in Schwann cells is associated with poor regeneration following long gap nerve repair using acellular nerve allografts (Saheb-Al-Zamani et al., 2013; Poppler et al., 2016; Hoben et al., 2018). However, in a recent review of the state-of-the-art for acellular approaches, the authors concluded that while they showed some potential, non-cellular constructs would likely need to incorporate a “recellularization step” to achieve comparable efficacy with the gold-standard autografts (Lovati et al., 2018).

In the current study, we aimed to develop a microtissue engineered living scaffold comprised of longitudinally-aligned Schwann cells as an alternative bridging strategy for peripheral nerve repair. The TE-BoB biofabrication protocol presented here would allow for the creation of a nerve graft that mimics the bundling of natural bands of Büngner that endogenously supports peripheral neuroregeneration. Similar to our previously reported microtissue engineered living scaffolds (Struzyna et al., 2015, 2017; Winter et al., 2016; Katiyar et al., 2018; O’Donnell et al., 2018), these Schwann cell constructs were constructed within a protective agarose hydrogel outer encasement with a collagen ECM inner core. Agarose was selected as the hydrogel for the micro-column due to several favorable biomaterial properties, such as biocompatibility, optical transparency, mass transport properties, relative inertness, and lack of adhesive ligands. In this application, the lack of adhesive ligands results in a hydrogel micro-column that provides geometric structure without inhibiting 3D cell/microtissue-mediated remodeling processes. During TE-BoB fabrication, Schwann cells extended processes and aligned longitudinally within the collagen substrate throughout the micro-column resulting in structural and phenotypic emulation of the bands of Büngner. For future translation, we demonstrated TE-BoBs fabrication a using clinically-applicable cell source, human Schwann cell-like cells derived from human gingiva-derived mesenchymal stem cells (GMSCs). These human TE-BoBs may present a clinically relevant solution to the morbidity associated with the commonly-used autograft and the limited efficacy of acellular bridging conduits/scaffolds.

After optimizing TE-BoB fabrication to produce longitudinally-aligned bundles of Schwann cells that emulate the structure and phenotype of bands of Büngner, we sought to test whether the TE-BoBs provided superior guidance of regenerating axons compared to Schwann cells in planar 2D culture or acellular agarose micro-columns containing only a collagenous matrix. Regenerating axons from MN and SN aggregates were found to precisely follow the dense longitudinal bundle of Schwann cells in the TE-BoBs, growing along and within the bundle itself. In contrast, the 2D controls revealed axonal extension from the aggregate in all directions. Additionally, acellular collagen-coated micro-column controls revealed limited longitudinal directionality, indicating some contribution of the angle of curvature of the micro-column itself to the directional guidance of axon growth, as we reported previously (Struzyna et al., 2015, 2018).

Axonal outgrowth length was also quantified to determine the regenerative potential of TE-BoBs relative to 2D and 3D controls. Neuron aggregates plated in aligned Schwann cell constructs resulted in extensive axonal outgrowth for both MNs and SNs. Faster SN axonal growth was found compared to MNs, supporting intrinsic differences in regenerative capacity between these neuronal subtypes (Cheah et al., 2017). Substantial sensory axon outgrowth was found within TE-BoBs, often spanning the entire length of the 5 mm construct by 4 days *in vitro*. Thus, the length of the micro-columns limited the maximum axonal outgrowth in this study. TE-BoBs resulted in 2× greater SN outgrowth compared to acellular control counterparts. For MN aggregates, the effects of TE-BoBs were more striking. Here, TE-BoBs also enhanced MN axonal outgrowth, and resulted in a remarkable 8x greater motor axon outgrowth relative to acellular collagen control micro-columns. These results indicate that the extensive growth is due to the presence of Schwann cells rather than the agarose micro-column and collagen ECM. The extent of axonal bundling was also assessed to determine the effect of longitudinally-aligned Schwann cells in TE-BoBs compared to constructs lacking Schwann cells. SN and MN axon bundles in TE-BoBs both were larger and covered a significantly greater portion of the lumen compared to bundles in the respective control constructs. This effect also varied based on neuronal subtype, as the average size and percent coverage of the axon bundles were approximately 3.8 and 1.5 times greater, respectively, for SN than MN bundles. Interestingly, a strong predictor for eventual motor recovery after nerve repair is early sensory reinnervation, suggesting that rapid sensory axon regeneration occurs first followed by motor axon regeneration (Jaquet et al., 2001). Our findings corroborate the clinical observations by showing sensory axons grow faster than motor neurons in our model of aligned Schwann cells, which likely recapitulate elements of pro-regenerative Schwann cells present after nerve injury.

Schwann cells also have a crucial role following nerve injury and during regeneration to preserve the proximal neuron health and regenerative capacity by providing multi-faceted neurotrophic support (Gordon, 2009). In the current study, Tuj1 protein expression within the aggregate region was assessed as a surrogate marker for neuronal health. Greater Tuj1 protein expression was found in MNs co-cultured with longitudinally-aligned Schwann cells compared to the collagen only control; whereas no differences were found in SNs co-cultured with longitudinally-aligned Schwann cells compared to the collagen only control. These findings suggest there may be preferential preservation of the MNs within the explant region in the presence of aligned Schwann cells. Interestingly, these findings corroborate other work suggesting SNs may be more resilient to extrinsic microenvironmental factors at acute time points following injury (Cheah et al., 2017; Maggiore et al., 2020).

Several biomaterial approaches have been proposed as potential replacements for autografts by mimicking structural guidance and/or neurotrophic support of aligned Schwann cells. These acellular approaches may act by mimicking features of aligned Schwann cells by selecting bioactive materials (e.g., spider silk) or by fabricating scaffolds from electrospun fibers or microgrooved polymer substrates to provide anisotropic cues (Sun et al., 2010; Daud et al., 2012; Kornfeld et al., 2016). While acellular approaches may enhance infiltration and regenerative capacity of host Schwann cells, TE-BoBs are designed to better represent autografts by serving as a preformed living scaffold for regenerating axons. Indeed, TE-BoBs are comprised of Schwann cells with similar morphology, protein expression, and function as native the bands of Büngner found in autografts and the distal nerve after injury. Similar to other preformed tissue engineered neural constructs, the longitudinally-aligned Schwann cells are densely bundled within a protective tubular hydrogel outer encasement that can be easily placed in a commercially-available nerve conduit for transplantation across nerve defects.

We selected both primary SN and MN aggregates for use in our *in vitro* neurite outgrowth assay to ascertain the potential of TE-BoBs to facilitate axon regeneration following nerve injury. Previous studies have demonstrated that aligned Schwann cell constructs improve neurite outgrowth *in vitro*, and this prior work provides a useful basis of comparison for our current findings. For example, the neurite growth rate from SNs within aligned Schwann cells has been reported to be 270 μm/day on electrospun polycaprolactone fiber scaffolds (Daud et al., 2012) and range from 178 to 270 μm/day on tethered aligned collagen (Phillips et al., 2005; Georgiou et al., 2013). In comparison, TE-BoBs achieved an average sensory neurite growth rate of 1,166 μm/day, indicating that the sensory growth rate achieved within our constructs is 4.3–6.5× faster than that achieved by alternative approaches. In addition, to the best of our knowledge, the current study is the first report demonstrating accelerated axonal outgrowth from MNs using aligned Schwann cell constructs. However, MN neurite outgrowth in non-aligned Schwann cell-seeded biomaterials has been reported to be 50 μm/day on 3 mm thick Matrigel (Hyung et al., 2015) and 61 μm/day on a collagen sponge co-cultured with fibroblasts (Gingras et al., 2008). In contrast, our study using 3D aligned Schwann cell constructs found MN outgrowth in TE-BoBs to be 653 μm/day, suggesting that TE-BoBs achieved axon growth rates that are 10.7–13.1× greater than these previous reports. Additionally, several studies using the NG108-15 cell line have reported increased neurite outgrowth in aligned constructs (35–334 μm/day) (Armstrong et al., 2007; Kingham et al., 2007; Sun et al., 2010; Daud et al., 2012); however, while not providing an ideal comparison as these are only “neuron-like” cells (Kowtha et al., 1993; Molnar and Hickman, 2007), these axon outgrowth rates are still below those achieved in TE-BoBs. Collectively, these stark improvements in axon growth rates for both sensory and motor neurons support the potential for improved PNS regeneration using TE-BoBs.

The accelerated axonal outgrowth induced by TE-BoBs may be partially due to the presence of NGFR p75 in the aligned Schwann cell constructs, which has been shown to facilitate axon pathfinding and regeneration in mice (Bentley and Lee, 2000; Tomita et al., 2007). However, regeneration is exponentially more complex than the signaling cascade of a single receptor. Relying on a single growth factor or receptor to promote regeneration is like trying to provide a solution to a complex problem with a one-word vocabulary. Tissue engineered constructs comprised of living cells are fluent in the language of cells. In contrast, acellular constructs may speak the equivalent of one word for each structural and/or soluble factor they contain, and acellular constructs are not “listening” to provide appropriately timed responses as is possible with living scaffolds. Indeed, TE-BoBs may be considered part of a broader class of living scaffolds that we have developed, which are designed to structurally and functionally mimic endogenous repair mechanisms relying on dynamic cell-to-cell interactions. For instance, we have engineered another glial-based construct comprised of aligned astrocytes that are designed to serve as a living scaffold for sustained neuronal replacement in the brain (Winter et al., 2016). By emulating the architecture and function of the endogenous glial tube in the rostral migratory stream, these constructs, described as a “tissue engineered rostral migratory stream” (TE-RMS) may redirect neuroblast migration and facilitate neuronal maturation (O’Donnell et al., 2018). Although both TE-BoBs and TE-RMS contain aligned glial cells fabricated using similar methodology, they interact with neurons very differently, promoting either axonal outgrowth or neuronal migration, respectively.

In addition to their therapeutic potential, TE-BoBs could also serve as an *in vitro* testbed for rapid, high throughput screening of mechanisms and efficacy of pro-regenerative strategies in a physiologically-relevant, 3D model of nerve regeneration. There is a growing demand across all science disciplines for tissue engineered 3D models which more closely mimic complex *in vivo* mechanisms, ultimately increasing translatable drug discovery and reducing the need for *in vivo* animal models (Nam et al., 2015; Vanderburgh et al., 2017; Rayner et al., 2018). Thus, the structural and phenotypical similarities between TE-BoBs and natural bands of Büngner suggest potential for future applications which require an anatomically- and physiologically-inspired pro-regenerative testing environment *in vitro*. For example, TE-BoBs may be useful to study various regenerative mechanisms, such as the role of c-Jun, a transcription factor that is considered the master regulator of the PNI response by governing the Schwann cell repair program, and the impact on neurite outgrowth following changes to pro-repair Schwann cell protein expression (e.g., GDNF, BDNF, NGF, or shh) (Arthur-Farraj et al., 2012).

There are several areas of TE-BoB optimization that will be explored in future studies. For instance, we were surprised to find that SNs extended neurites through the full length of the TE-BoBs used in the current experiments; since we can fabricate significantly longer TE-BoBs, future studies will investigate the maximal limits of axon growth facilitated by TE-BoBs. In addition, further optimization studies may be warranted to fabricate TE-BoBs specifically designed to accelerate motor or sensory axon outgrowth. Moreover, although agarose is a relatively inert biomaterial which would likely result in minimal *in vivo* host response, it exhibits slow rates of degradation and resorption *in vivo*. The modular fabrication methodology readily allows for the use of alternative hydrogel micro-columns and ECM constituents depending on the scientific question or the specific application. Therefore, it may be useful to investigate alternative encapsulation strategies, such as agarose composite hydrogels, such as agarose and gelatin, to further enhance degradation, resorption, biocompatibility, provide complimentary release of drugs or neurotrophic supplements, or to fine-tune other physical properties.

In this study, Schwann cells plated in the micro-column rapidly self-assembled into a longitudinal orientation. By tuning these physical properties, it may be possible to inhibit the self-assembly process and further investigate whether unaligned Schwann cells in a 3D micro-column increase neurite length/outgrowth. Notably, a previous study using genetic lineage tracing in a mouse model has shown that aligned Schwann cells are able to remyelinate regenerating axons *in vivo* (Gomez-Sanchez et al., 2017). Indeed, previous studies have reported myelination occurs around neurons co-cultured with primary rodent Schwann cells at later time points, often around 28 days *in vitro*, with the addition of ascorbic acid (Callizot et al., 2011). Therefore, it is possible that the axons extending within the aligned Schwann cell constructs may eventually undergo myelination.

Also, the current study generated TE-BoBs using both primary rodent Schwann cells and human GMSC-derived Schwann cells, however, future work will focus on these Schwann cells derived from easily accessible human GMSCs that are available throughout adulthood in humans (Zhang et al., 2018a,b). Additional investigation of TE-BoB fabrication using these human stem cells as well as neurite outgrowth and eventual myelination using human neurons – from allogeneic or even autologous sources – may provide mechanistic insights into the translational potential of TE-BoBs. Lastly, this biofabrication process is readily scalable, enabling the creation of longer lengths for testing in long gap PNI models that are greater than the critical nerve gap length of rats (2 cm) and humans (5 cm). These modifications would further advance TE-BoBs as an effective peripheral nerve repair strategy that mimics key advantages of the gold standard autograft repair, yet eliminates several of the shortcomings of current repair strategies.

The ultimate TE-BoB repair strategy for PNI may involve implanting several aligned Schwann cell constructs within a larger nerve conduit—for instance, one TE-BoB per fascicle—to provide living bridges spanning a segmental nerve defect. Indeed, it would be trivial to build multi-lumen constructs for TE-BoB fabrication, or even to stack multiple versions of the current TE-BoBs within a nerve guidance wrap, for testing in larger caliber nerves. We postulate that these TE-BoBs would augment endogenous mechanisms of regeneration by providing preformed bands of Büngner in cases where the gap lengths are too great for host Schwann cells to infiltrate and fill. Direct contact with the proximal side of the nerve defect will enable axons to extend through the engineered aligned Schwann cells and efficiently transverse the gap to ultimately reach the endogenous bands of Büngner within the distal nerve sheath that provide targeted axon guidance to appropriate sensory and/or muscle targets. Given the promising results of this *in vitro* study, we will proceed to test the efficacy of the TE-BoB repair strategy using appropriate *in vivo* models of PNI.

## Conclusion

We demonstrated the development and validation of the first tissue engineered bands of Büngner (TE-BoBs) comprised of three-dimensional, longitudinally aligned bundles of pro-regenerative Schwann cells. TE-BoBs were biofabricated based on a biomaterial guided cell self-assembly scheme using either rat primary Schwann cells or human stem cell derived Schwann cells. Functional testing using *in vitro* neurite outgrowth assays revealed that TE-BoBs directly facilitated and accelerated longitudinal axonal outgrowth from both primary motor and sensory neurons as compared to that measured in 2D and 3D control groups. Moreover, TE-BoBs achieved motor axon and sensory axon growth rates that were at least 10.7× and 4.3× faster, respectively, than rates achieved by alternative Schwann cell-mediated strategies. These self-assembled, aligned glial constructs represent a novel approach utilizing microtissue engineering strategies that specifically recapitulate 3D biological “living scaffolds” found *in vivo* to direct axonal outgrowth. Furthermore, given that long gap PNIs often result in insufficient axonal growth leading to failed muscle innervation, future repair strategies that can overcome this barrier have significant clinical relevance. With further development, these TE-BoBs may serve as implantable microtissue that can supplement or replace the use of autograft techniques to accelerate axon outgrowth across segmental defects and thereby enhance peripheral nerve regeneration and functional recovery.

## Data Availability Statement

The raw data supporting the conclusions of this article will be made available by the authors, without undue reservation.

## Ethics Statement

Human gingival tissues were obtained as remnants of discarded tissues from healthy human subjects aged from 20 to 40 years old, who underwent routine dental procedures. Informed consents were obtained from all subjects and all procedures were performed under the approved Institutional Review Board (IRB) protocol at the University of Pennsylvania.

## Author Contributions

DKC conceived the study and provided the experimental design. KP and KH fabricated TE-BoBs and completed *in vitro* assays. KP and JB conducted the *in vitro* histological assessments and statistical analyses. KH, EP, and JO provided technical assistance with fabrication and quantification, and assisted with figure preparation. QZ and AL provided the human gingiva-derived Schwann cell-like cells. KP, JB, and DKC prepared the final manuscript. All authors provided critical feedback on the manuscript.

## Conflict of Interest

The authors declare that the research was conducted in the absence of any commercial or financial relationships that could be construed as a potential conflict of interest.
